# Suppression of Plant Resistance May Be a Common Trait Among Adapted Herbivores

**DOI:** 10.1002/ece3.71706

**Published:** 2025-07-14

**Authors:** Jéssica Teodoro‐Paulo, Carlos Fernandes, Lin Dong, Sara Magalhães, Alison B. Duncan, Juan M. Alba, Merijn R. Kant

**Affiliations:** ^1^ cE3c—Centre for Ecology Evolution and Environmental Changes & CHANGE—Global Change and Sustainability Institute, Faculty of Sciences University of Lisbon Lisbon Portugal; ^2^ Institute for Biodiversity and Ecosystem Dynamics University of Amsterdam Amsterdam the Netherlands; ^3^ Institut Des Sciences de l’Évolution University of Montpellier Montpellier France; ^4^ Institute for Mediterranean and Subtropical Horticulture La Mayora (IHSM), CSIC‐UMA Málaga Spain

**Keywords:** effector 84, effector diversity, immune suppression, intraspecific variation, salivary proteins, *Tetranychus urticae*

## Abstract

Herbivores have evolved distinct strategies to cope with plant defenses prior to becoming a pest. Some evolved resistance to toxic compounds; others suppress host toxin production. These traits seem to co‐occur within herbivore species, particularly among spider mites, which are major pests in many crops. The intraspecific variation within the spider mites is a model for adaptive pest evolution on crops such as tomato. Empirical data collected from nonsolanaceous wild host plants suggest that natural populations predominantly comprise individuals capable of inducing tomato defenses, while a small proportion suppress these defenses. Additionally, resistant individuals are observed only rarely within these populations. This study aimed to investigate the presence of these traits within populations adapted to tomato plants. Here, *Tetranychus urticae* populations sampled from tomato at three field sites were compared. To discriminate between mites that induce/suppress defenses and to assess their degree of resistance, the magnitude of induced defenses was measured and aligned with oviposition data. The expression of effector 84 was also assessed to determine whether its magnitude of expression is a good predictor of suppression with the magnitude of suppression. Surprisingly, we observed that suppression is the dominant phenotype in mite populations collected from field‐grown tomatoes. Our results suggest that suppression may be rare only at the beginning of an herbivore's adaptive trajectory after colonization of a novel host but may rapidly become common due to natural selection. This suggests the possibility that suppression potentially represents a prevalent phenotype among host‐adapted herbivores and, consequently, among pests.

## Graphical abstract

The intraspecific variation within the spider mites is a model for adaptive pest evolution on crops such as tomato. Here, *Tetranychus urticae* populations sampled from tomato at three field sites were compared, revealing that suppression is the dominant phenotype in mite strains collected from field‐grown tomatoes. It seems that suppression may be rare only at the beginning of an herbivore's adaptive trajectory after colonization of a novel host but may rapidly become common due to natural selection, implying that suppression may be a common phenotype among host‐adapted herbivores and consequently among pests.

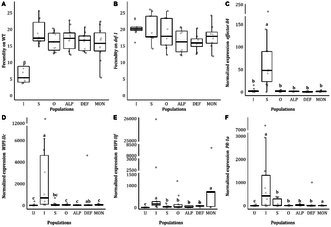

## 1 Introduction

Plants are equipped with constitutive and inducible defenses to deter herbivores (Agrawal and Karban 1999). While constitutive defenses traits are premade, like physical barriers or toxins, inducible defenses are established after herbivore attack (reviewed in Vega‐Muñoz et al. 2020) and can include reinforcement of constitutive defenses. This inducible response consists of a collection of defense programs, regulated primarily by the plant hormones jasmonic acid (JA) (Wasternack and Hause 2013) and salicylic acid (SA) (Vlot et al. 2009), and aims at reducing the quality of the plant as food. The JA pathway is mostly responsible for defense responses against necrotrophic pathogens and chewing or cell content‐feeding herbivores (Walling 2000), while the SA pathway is mostly induced by biotrophic pathogens (Glazebrook 2005) and phloem‐feeding insects (Kaloshian and Walling 2005). However, the SA pathway can also be triggered by herbivory, leading to the induction of pathogen resistance (PR) genes (e.g., in aphids, Moran and Thompson 2001; spider mites, Alba et al. 2015). This can include the synthesis of detrimental proteins, for example, by the induction of proteinase inhibitors that reduce herbivore protein digestion (Walling 2000), which thereby decrease herbivore development (Karban and Myers 1989), and population growth, promoting plant fitness (Zavala and Baldwin 2004; Turrà et al. 2020). In response to selection pressure by these defensive mechanisms, some herbivore species (e.g., arthropods and nematodes) have evolved counter adaptations that either enable them to neutralize (detoxify) defensive metabolites (Després et al. 2007; Heckel 2014; reviewed in Groen and Whiteman 2022) or suppress the plant's defense metabolism (e.g., reviewed in Kant et al. 2015).

Metabolic resistance can be established through detoxification (i.e., metabolic modification, Cianfrogna et al. 2002; degradation, Heckel 2014 and secretion), sequestration (e.g., Bowers 2022), or the evolution of target‐site insensitivity (Berenbaum 1986). Metabolic resistance can be efficient for several herbivore species living on a range of hosts with similar secondary chemistry. However, for generalists, stacking different modes of metabolic resistance to cope with a wider variety of defensive substances may be costly and thus readily erode under natural selection (Ali and Agrawal 2012; Blaazer et al. 2018). However, it was also shown that some generalists cope with their variable hosts by interfering with the defense pathways of different plant species (Kant et al. 2015). This interference often comes down to suppression of defenses (reviewed in Blaazer et al. 2018) and is characterized by a reduction in induced toxin accumulation (e.g., reduced nicotine accumulation by feeding caterpillars in the leaves of tobacco, Musser et al. 2002) and induced defense gene expression (Kant et al. 2004) either to intermediate levels (Alba et al. 2015) or even to levels below those of unattacked control plants (Sarmento, Lemos, Bleeker, et al. 2011; Sarmento, Lemos, Dias, et al. 2011; Schimmel et al. 2018). This suppression boosts the performance of the suppressor (Sarmento, Lemos, Bleeker, et al. 2011; Sarmento, Lemos, Dias, et al. 2011) but also can boost the performance of nearby nonsuppressors residing on the same plant (Kant et al. 2004), and suppressors can downregulate defenses across a diverse range of hosts (Paulo et al. 2018). Suppression of plant defenses is a well‐known trait among phytopathogens and is established via molecules, often referred to as “effectors,” secreted into the host during the early and later stages of the infection (reviewed in Jones et al. 2022). Data on the functionality of effectors across multiple hosts is still rare, but some host plants have evolved mechanisms to detect the activity of effectors and by‐pass their suppression effectively, turning the effector into an elicitor of plant defenses (reviewed in Kallure et al. 2022). Although effectors are associated with defense suppression, herbivores that induce defenses may also possess them (Villarroel et al. 2016) and thus effector‐mediated induction/suppression depends on the presence of effector targets in the host as well as the host's ability to detect and respond to the collective of secreted substances (Cui et al. 2022).

*Tetranychus urticae* is a generalist spider mite species (Helle and Sabelis 1985) and a model herbivore for studying plant–herbivore interactions (Blaazer et al. 2018). When feeding on tomato plants ( *Solanum lycopersicum* ), most of the *T. urticae* strains studied induce JA‐ and SA‐dependent plant defenses leading to decreased herbivore performance and survival (e.g., Kant et al. 2008; Sarmento, Lemos, Bleeker, et al. 2011; Sarmento, Lemos, Dias, et al. 2011; Alba et al. 2015; Godinho et al. 2016). As such, most of the populations of this species are believed to be susceptible to the tomato JA and SA defenses that are induced through feeding (susceptible‐inducer populations). However, some strains can resist the plant defenses they induce (resistant‐inducer populations, Kant et al. 2008). It was shown that the *T. urticae* genome has multiple gene families implicated in xenobiotic metabolism, whose magnitude of expression changes markedly when adapting to new environments (Zhurov et al. 2014; Wybouw et al. 2015). This suggests that traits related to metabolic resistance may be favored by natural selection during adaptation to a novel host (Van Leeuwen and Dermauw 2016; Blaazer et al. 2018). Nevertheless, adaptation to a novel host also appeared to improve the population's ability to suppress defenses (susceptible‐suppressor populations, Wybouw et al. 2015). The ability to suppress tomato defenses was found to exist in low frequencies in natural *T. urticae* populations while in another species, *T. evansi* , it appeared to be a fixed species trait (Kant et al. 2008; Sarmento, Lemos, Bleeker, et al. 2011; Sarmento, Lemos, Dias, et al. 2011; Alba et al. 2015). Even since the discovery of defense suppression as an alternative to xenobiotic resistance in mites, researchers have speculated about the role of these traits during adaptation to novel hosts and how they relate to herbivores having a generalist or specialist life styles (Blaazer et al. 2018). Defense suppression by spider mites has been attributed to salivary proteins called effectors and especially effector family 84 (Tu84 for *T. urticae* and Te84 for *T. evansi* in Villarroel et al. 2016; Schimmel et al. 2017; Liu, Chafi, et al. 2020). Here we will refer to these as “*effector 84*.” The expression of *effector 84* is plastic and changes in response to the presence of con‐ and heterospecific competitors (Schimmel et al. 2017), to light/dark conditions (Liu, Chafi, et al. 2020) and differs across mite life stages and sexes (Liu, Legarrea, et al. 2020).

Previous studies found that natural populations of spider mites collected on nonsolanaceous host plants, such as European spindle tree (*Euonymus europea*), deadnettle ( *Lamium album* ), and castor oil ( *Ricinus communis* ), exhibit phenotypic diversity. These populations predominantly comprise individuals that induce and are susceptible to tomato defenses, while a smaller portion suppresses these defenses, with occasional occurrences of inducer and resistant individuals (Kant et al. 2008; Alba et al. 2015). As such, in this study, we asked if such diversity can be found in natural populations living on field tomatoes grown using organic or biological farming practices, as it was shown that nonsuppressor mite strains from bean can evolve defense suppression on tomato after 30 generations (Wybouw et al. 2015). Hence, we aimed to understand if mites naturally occurring on field‐grown tomatoes display this trait as well, and if the magnitude of expression of the *effector 84* correlates with the level of defense suppression. We hypothesized that (i) spider mites residing on tomato in the field will be adapted to the defenses of these plants and thus harbor primarily defense‐resistant individuals, individuals that can suppress defenses, or a mixture, (ii) defense suppression correlates with the expression of *effector 84*, and (iii) inducer and suppressor individuals would comprise different phylogenetic clades on the basis of their *effector 84* genotype. As such, we aimed to phenotype three mite populations collected from field‐grown tomatoes for (i) their ability to resist or suppress tomato defenses, (ii) the correlation between *effector 84* expression and the magnitude of induced defenses, and (iii) a phylogenetic analysis of *effector 84*.

For this, we compared three *T. urticae* populations sampled from tomato at three field sites and also an outbred population created from these via controlled crosses. We then assessed their fecundity on tomato plants with (wildtype, WT) and without (*def‐1*) inducible JA defenses, as well as the magnitude of JA defenses they induce in WT plants, to discriminate between resistant types (high induction and high performance) and those that can suppress defenses (low induction, high performance). We then aligned these data with data on variation in mitochondrial DNA cytochrome oxidase I (*COI*) and *effector 84* (which can suppress JA defenses), in order to compare genetic diversity patterns among mite lines that induce or suppress defenses. We found that suppression is the dominant phenotype in mite populations collected from field‐grown tomatoes and that inducers and suppressors predominantly cluster in distinct *effector 84* clades.

## 2 Material and Methods

### 2.1 Plants

Tomato plants ( *Solanum lycopersicum* L.), of the varieties Castlemart (both *WT* and *defenseless‐1*, *def‐1*) and Moneymaker, and bean plants ( *Phaseolus vulgaris* L. cv Speedy) were germinated and grown (soil—Siro interior, Siro, Mira, Portugal) in a climatic chamber (photoperiod of 16:8 h, 25°C:18°C, day: night, 50%–60% relative humidity (RH)) for 28 and 10 days, respectively. On *def‐1* tomato plants, JA defenses cannot be induced (Bergey et al. 1996; Howe et al. 1996; Li et al. 2002), making them a great tool to assess the effect of JA defenses on spider mites. Experiments were carried out in a climatic chamber (photoperiod of 16:8 h, 23.5°C ± 2°C, 70% RH), to which *WT* and *def‐1* tomato plants (cv Castlemart) were transferred 1 day before the beginning of the experiment.

### 2.2 Spider Mites

*Tetranychus urticae* was sampled in the field at three locations, from here on referred to as three mite populations (ALP, DEF, and MON). The populations were collected in tomato organic farms in Portugal in 2017 (more detailed information on collection site, time of the year, and plant variety can be found in Godinho et al. 2020) and were subject to a heat shock treatment for 42 days to remove endosymbionts such as *Wolbachia* (Godinho et al. 2020). These symbiont‐free populations were then used to create an outbred laboratory population (Outbred) by performing interpopulation, two‐way (i.e., female 1 × male 2 and female 2 × male 1) controlled crosses to normalize over‐representation of genotypes. The outbred population started with more than 100 individuals (> 50 males and > 50 females) of each cross combination (Godinho et al. 2020). As such, since the outbred population represents a genetic mixture of the original populations due to interbreeding, studying this population allows for a better understanding of how the introduction of new genetic combinations through interbreeding influences traits expression and genetic diversity.

All field and outbred populations were reared on detached tomato leaves (cv Moneymaker) with the petioles submerged in water.

All populations were maintained in a climatized room (photoperiod of 16:8 h, 23.5°C ± 2°C), inside 4‐L boxes with a mesh lid to allow airflow.

#### 2.2.1 Benchmark Populations and Biomarker

*Tetranychus urticae* Santpoort‐2 (“KMB” in Kant et al. 2008, “Santpoort‐2” in Alba et al. 2015) was used as defense‐inducer benchmark. For the defense‐suppressor benchmark, *T. evansi* Baker & Pritchard Viçosa‐1 (Alba et al. 2015) was used, due to its inherent characteristic to consistently suppress tomato plant's defenses, which presents a marked contrast to the general scarcity of fully suppressed populations within *T. urticae* (up to this study), offering a valuable reference point for evaluating and understanding defense manipulation mechanisms among spider mite species. These populations have been widely used in studies of tomato–mite interactions (e.g., Sarmento, Lemos, Bleeker, et al. 2011; Sarmento, Lemos, Dias, et al. 2011; Villarroel et al. 2016; Schimmel et al. 2017; Liu, Chafi, et al. 2020; Liu, Legarrea, et al. 2020) and are well‐characterized inducer and suppressor populations, respectively (Alba et al. 2015). This inherent characteristic of *T. evansi* to consistently suppress tomato plant defenses presents a marked contrast to the general scarcity of fully suppressed populations within *T. urticae* (up to this study). In the absence of a good benchmark or reference control for evaluating the degree of tomato defense suppression within *T. urticae* species, we used *T. evansi* Baker & Pritchard Viçosa‐1. The defense‐inducer benchmark was reared on detached bean plants (cv. Speedy), and the defense‐suppressor benchmark was reared on detached tomato leaves (cv Castlemart). Plants infested with these benchmark populations and uninfested plants were used as controls across all assays.

*Tetranychus urticae* Santpoort‐2 can also be used as a biomarker of JA‐induced defenses since this population is susceptible to JA defenses (Kant et al. 2008). When on a plant preinfested with an inducer mite, the fecundity of this biomarker is lower than on an uninfested plant or than on a plant preinfested by a suppressor population (Kant et al. 2008; Sarmento, Lemos, Bleeker, et al. 2011; Alba et al. 2015). As such, this is a perfect biomarker to disentangle between resistant (i.e., inducer/resistant) and suppressor strains.

### 2.3 Fecundity of *T. urticae* From Field‐Collected Populations on WT and *Def‐1*

We assessed the fecundity of each of the *T. urticae* populations when JA defenses are induced (using WT) and in the absence of these defenses (using *def‐1*). To this end, 30 mated females (15 ± 1 day old) from each *T. evansi* and *T. urticae* population were placed on the same, nonterminal leaflet of a fully expanded leaf of whole WT or *def‐1* tomato plants (Day 0, Figure S1). Mite dispersal was prevented by isolating the adaxial surface of this leaflet with a 1:1 mix of entomological glue (Tanglefoot, The Scotts Company LLC, OH, USA) and lanolin (Sigma‐Aldrich, St. Louis, MO, USA), which was distributed around the adaxial edge of the leaflet. For each population and replicate, individual whole plants were used. Following 4 days of infestation (Day 4), the number of surviving females on the leaflet and the number of eggs laid were counted. With these two measures, we calculated fecundity per female by using: [total eggs]/[(alive females + total females)/2]. This equation accounts for differential female mortality across treatments, which is measured at the end of the assay and thus enables a more accurate representation of per capita fecundity (Teodoro‐Paulo et al. 2023). There were 10–11 and eight to nine replicates for each population on WT and *def‐1* whole plants, respectively. Assays on WT and *def‐1* whole plants were not performed simultaneously due to logistical constraints.

### 2.4 Fecundity of the Biomarker Strain on Leaflets That Had Been Preinfested by Mites From Field‐Collected Populations

Similar to what was described above, we placed biomarker mites ( *T. urticae* Santpoort‐2) on whole WT plants that had previously been infested, for 4 days, with our field and outbred populations. We first infested tomato leaflets with mites from a field‐collected population for 4 days and then removed all mites and eggs to clean the leaflet. Cleaned leaflets were then reinfested with three biomarker individuals (15 ± 1 days old). Biomarker survival and the number of eggs were then recorded after 48 h (i.e., 6 days post the primary infestation). There were five to 11 replicates for each population.

### 2.5 Induction of Tomato Defense Genes by the Field‐Collected Spider Mites and Expression Levels of Their *Effector 84*

We analyzed transcript accumulation of genes implicated in JA defenses (*Proteinase Inhibitor IIc, WIPI‐IIc* and *Proteinase Inhibitor IIf, WIPI‐IIf*; Alba et al. 2015) and SA defenses (*Pathogenesis‐related protein 1a, PR‐1a*; Alba et al. 2015) in WT plants infested with mites from the field‐collected and outbred populations. We also analyzed transcript accumulation of *effector 84* from these same mites by using the same RNA that had been collected for assessing the expression of the plant defense genes. *Ribosomal protein 49* (*RP49*) and *Actin* were used as housekeeping genes for spider mites and tomato plants, respectively (see Table S1 for primer sequences).

Material for collecting mite and plant RNA was prepared as follows: from infested leaflets, we cut out the part of the infested lamina within the glue and lanolin barrier and this was flash‐frozen in liquid nitrogen and stored at −80°C. For each treatment, we obtained four to 11 replicates.

Total RNA was isolated from leaves using a protocol adapted from Verwoerd et al. (1989). Our protocol differs from it in that: (i) we used phenol at room temperature (RT) instead of hot phenol (heated to 80°C) and (ii) completed the 5‐min sample incubation step at RT instead of 80°C. Next, 2 μg of RNA were DNAse‐treated with Ambion Turbo DNA‐free kit (Thermo Fisher Scientific, Waltham, Massachusetts, USA) and cDNA was synthesized with RevertAid H Minus Reverse Transcriptase (Thermo Fisher Scientific, Waltham, Massachusetts, USA). 1 μL of 10‐times‐diluted cDNA was used as the template for a 20 μL quantitative reverse‐transcriptase polymerase chain reaction (RT‐qPCR) using the CFX96 Real‐Time system (Bio‐Rad, Hercules, California, USA) with SsoFast EvaGreen Supermix (Bio‐Rad, Hercules, California, USA). Transcript accumulation was normalized using the ΔC_*t*_ method (Alba et al. 2015). To produce a graphical representation that is easier to read in a data‐neutral manner, all values were divided by the lowest average in the graph.

### 2.6 Statistical Analysis

All statistical analyses were performed with the software R (version 4.2.2, R Development Core Team 2022, Chichester, UK).

To investigate whether fecundity (at Day 4) varied among populations in WT or in *def‐1* host plants, generalized mixed linear mixed models (GLMM) with a normal error structure (lmer, lme4 package, Bates et al. 2015) were performed, since normality was met (WT: Shapiro–Wilk test: *p* = 0.180; *def‐1*: Shapiro–Wilk test: *p* = 0.219), and variances were homogeneous. Each model, one for each plant type (WT or *def‐1*) included *population* (i.e., *T. urticae* Outbred, ALP, DEF, MON), as fixed explanatory variables, and *block* (defined as the group of experimental treatments performed in the same day, and accounts for variability that are not of primary interest) as a random variable. This was repeated including the benchmark controls (WT: Shapiro–Wilk test: *p* = 0.091; *def‐1*: Shapiro–Wilk test: *p* = 0.659, and homogeneous variances for both datasets).

To evaluate whether the fecundity of the biomarker, 6 days post primarily infestation, was affected by preinfestation with the *T. urticae* populations, we performed a GLMM model with a normal error structure (lmer, lme4 package) including *population* as a fixed explanatory variable and *block* as a random variable. This is because normality was met (Shapiro–Wilk test: *p* = 0.794) and variance was homogeneous.

The normalized transcript accumulation of *effector 84*, 4 days after infestation, was analyzed by performing a GLMM assuming a Gamma distribution and a log‐link function (glmmTMB, glmmTMB package, Brooks et al. 2017), since normality was not met (Shapiro–Wilk test: *p* < 0.001), but variances were homogeneous. The model included *population* as a fixed explanatory variable and *block* as a random factor.

To assess the impact of mite feeding on the expression of plant marker genes, independent GLMMs assuming a Gamma distribution and a log‐link function (glmmTMB, glmmTMB package, since normality was not met, Shapiro–Wilk test: *p* < 0.001, but variances were homogeneous) were performed for the genes *WIPI‐IIc, WIPI‐IIf*, and *PR‐1a*. All models included *population*, *time point* (i.e., 4 days post infestation—4dpi—or after the second infestation with the biomarker, i.e., 6 days post infestation—6 dpi) and their interaction as fixed explanatory variables and *block* as a random variable. The models included the benchmark controls.

When significant differences were found, multiple comparisons were performed using estimated marginal means (emmeans, emmeans package) (Lenth 2022), and the *p* values corrected using the false discovery rate (FDR) method (*α* = 0.05) (Benjamini and Hochberg 1995). Cytochrome oxidase 1 (*COI*) and *effector 84* sequencing and assembly.

The genomic DNA (gDNA) of single adult females of the field‐grown and laboratory populations (Table 1) was extracted by first crushing the specimens, previously frozen at −80°C, with a plastic pestle and adding 100 μL of 5% Chelex solution (Chelex 100 sodium form, Sigma‐Aldrich, St. Louis, USA) and 5 μL proteinase‐K (20 mg/mL). The samples were incubated at 56°C for 60 min, followed by denaturation at 95°C for 8 min (Walsh et al. 1991). Amplification of *COI* (seven to 14 separate individuals per population) and *effector 84* (eight to 13 separate individuals per population; see primers in Table S1) was performed using endpoint PCR and the presence of PCR product was confirmed on ethidium bromide‐stained agarose gels. The remaining PCR products (1× diluted) were sent to Eurofins Genomics for Sanger sequencing.

**TABLE 1:** Collection records of the *T. urticae* populations used for *COI* and *effector 84* sequencing.

Population	Original population	Laboratory host	Field host	Location	Year	References
ALP	ALP	*Solanum lycopersicum* (cv. Moneymaker)	*Solanum lycopersicum*	Alpiarça, Portugal	2017	Godinho et al. (2020)
DEF	DEF	*Solanum lycopersicum* (cv. Moneymaker)	*Solanum lycopersicum*	Lisboa, Portugal	2017	Godinho et al. (2020)
MON	MON	*Solanum lycopersicum* (cv. Moneymaker)	*Solanum lycopersicum*	Montemor‐o‐Novo, Portugal	2017	Godinho et al. (2020)
Outbred	Outbred	*Solanum lycopersicum* (cv. Moneymaker)	Laboratory created population	NA	2018	Godinho et al. (2020)
Santpoort‐1^a^	KMT	*Solanum lycopersicum* (cv. Castlemart)	*Euonymus europea*	Santpoort, Netherlands	2001	Kant et al. (2008)/NCBI bioproject PRJNA78687
Santpoort‐2^b^	KMB	*Phaseolus vulgaris* , cv. Speedy	*Euonymus europea*	Santpoort, Netherlands	2001	Kant et al. (2008)
SN^a^	NA	*Solanum lycopersicum* (cv. Castlemart)	*Solanum nigrum*	Málaga, Spain	2010	Sato et al. (2014)
London_NL^a^	London	*Phaseolus vulgaris* , cv. Speedy	NI	Ontario, Canada	NI	Grbić et al. (2011)

Abbreviations: NA, nonapplicable; NI, no information available.

^a^ Defense‐inducer benchmark and biomarker strain.

^b^ Well documented populations in literature, which serve, in the present study, as a benchmark. more informations about this populations can be found in Kant et al. (2008), Sato et al. (2014) and Grbić et al. (2011), respectively.

The sequences were assembled and edited (i.e., sequence peaks were confirmed and corrected when needed) in SeqMan Pro 14 (version 14.1.0 (118), DNASTAR Lasergene 14, Madison, Wisconsin, United States) and aligned using MUSCLE (Edgar 2004) in MEGA (version 11, Tamura et al. 2021). The *COI* alignment consisted of 115 sequences (385 bp), from which 35 were from GenBank (34 of *T. urticae* and one of *T. evansi* as an outgroup). From these, haplotypes were obtained using the DNAcollapser tool of Fabox (Villesen 2007, Table S3).

The reference *effector 84* gDNA sequences can be found in OrcAE (Sterck et al. 2012) as tetur01g01000 ( *T. urticae* London and *T. urticae* Montpellier genome). The allele and protein variants obtained for *effector 84* can be found in Table S2. The ORF in the *effector* 84 gDNA spans two exons: the first of 42 nucleotides and the second of 697 nucleotides separated by an intron 85 nucleotides long. We amplified 635 nucleotides from the second exon (see Figure S2). The *effector 84* alignment consisted of 103 sequences (635 bp, which, based on the gene model of Villaroel et al. 2016, corresponds to nucleotides 55 to 690), from which three were from GenBank (two of *T. urticae* and one of *T. evansi* as an outgroup—the latter with 637 bp). Note that since female mites are diploid, we sometimes obtained two haplotypes per individual. To resolve this, we implemented a Bayesian statistical method for phasing both haplotypes using PHASE v2.1 (Stephens et al. 2001) in DNAsp v5 (Librado and Rozas 2009).

### 2.7 Sequence Analysis

For *effector 84*, DNA polymorphisms were analyzed using DNASP 4.0 (Rozas et al. 2003). As a measure of DNA polymorphism within populations, five parameters were estimated: the number of polymorphic/segregating sites (*S*), the total number of mutations (*Eta*), the number of haplotypes (*h*), the haplotype diversity (*H*_d_, a measure of the frequencies and number of haplotypes among individuals, varying between 0 and 1, Nei 1987), and the nucleotide diversity (*π*, average weighted sequence divergence between haplotypes, varying between 0 for no divergence to 10% for very deep divergences, Tajima 1983). Note that this analysis was performed for the complete sequence dataset, but also for individual populations (i.e., ALP, DEF, MON, outbred, London_NL).

### 2.8 Phylogenetic Analysis

We used PartitionFinder2 (Lanfear et al. 2016) and ModelFinder (Kalyaanamoorthy et al. 2017) to select the best partition arrangement and the most suitable evolutionary substitution model (*COI*: HKY + F + I for subset1 = 1–385\3 3–385\3, and HKY + F for subset2 = 2–385\3; *Tu84*: GTR + F + I + G4 for subset1 = 1–638\3 2–638\3 3–638\3). Bayesian Inference phylogenies were inferred using MrBayes 3.2.6 (Ronquist et al. 2012) (two parallel runs, 20 million generations), in which the initial 25% of sampled data were discarded as burn‐in. All the analyses were run using the PhyloSuite version 1.2.2 desktop platform (Zhang et al. 2020). With the trees obtained from MrBayes, we computed 70% majority‐rule consensus trees using SumTrees using SumTrees (Sukumaran and Holder 2010) from the Dendropy library (version 4.1.0, Sukumaran and Holder 2010).

*Tu84* sequences were translated following the gene model of Villaroel et al. (2016). For the protein analysis, the best phylogenetic model was estimated in MEGA 11. Then, a maximum likelihood phylogenetic analysis with 1000 bootstraps, using the LG + G model with gamma shape = 0.67, was performed, and a consensus tree with bootstraps higher than 50% was obtained using MEGA. The phylogenetic trees were edited in iTOL version 6.5.8 (Letunic and Bork 2021).

### 2.9 Accession Numbers

The sequence data from this article are available at NCBI website (https://www.ncbi.nlm.nih.gov) and can be found under the following accession numbers: *Tu84*, OQ472023–OQ472057; *COI*, OQ510005–OQ510008.

## 3 Results

### 3.1 Fecundity of *T. urticae* From Field‐Collected Populations on WT and *Def‐1*

**FIGURE 1:**
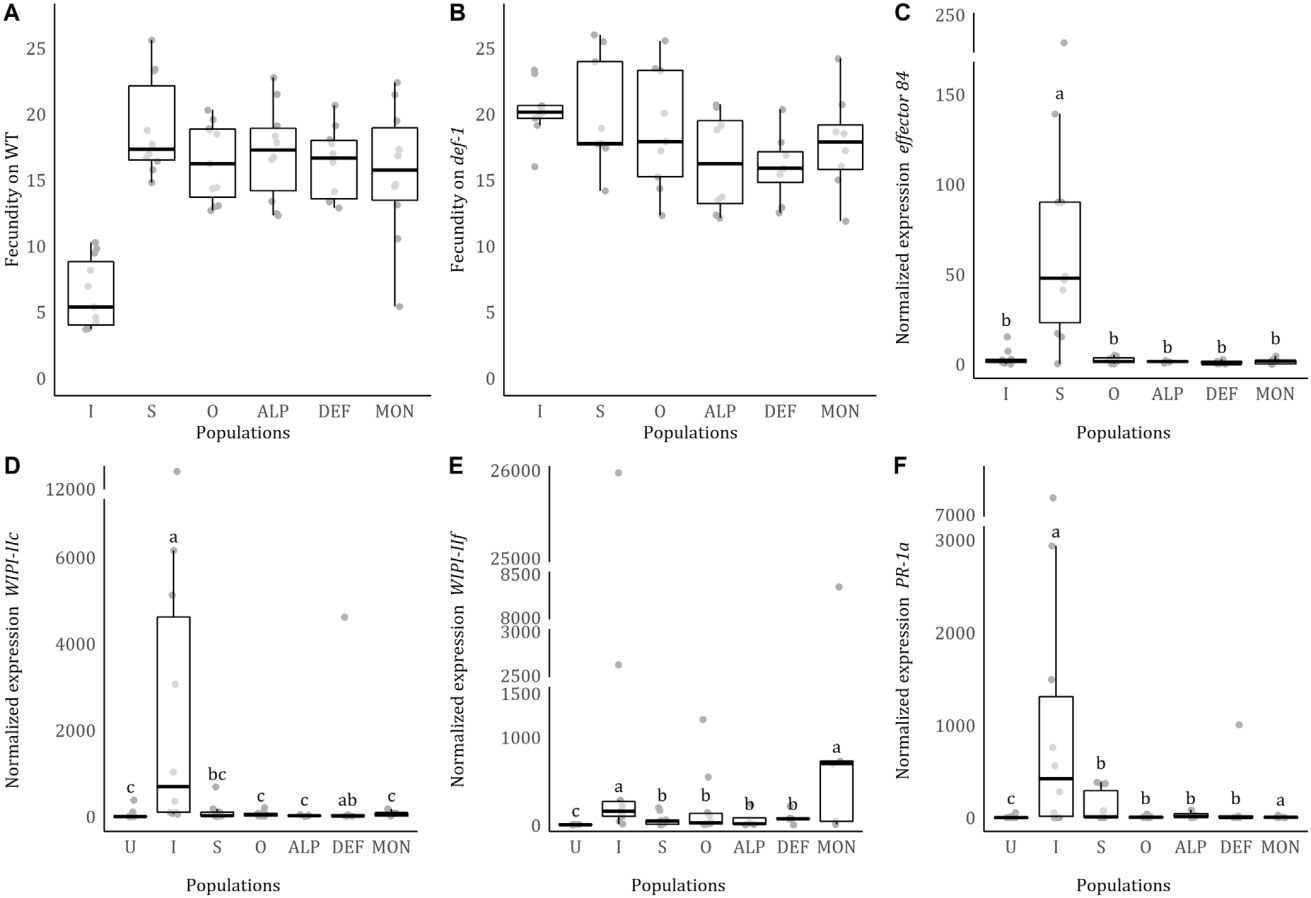
Phenotypic characterization of the field‐collected and the outbred populations. We measured fecundity of each population in the (A) presence (WT, white) and (B) absence (*def‐1*, gray) of JA defenses after 4 days of infestation, and transcript levels of (C) salivary *effector 84*, JA‐related defenses (D) *WIPI‐IIc*, (E) *WIPI‐IIf*, and SA‐related defenses (F) *PR‐1a* at 4 dpi. Each circle represents individual data points (*n* = 8–11). Different lowercase letters denote statistical differences between populations according to multicomparison analysis performed using estimated marginal means. The benchmarks are denoted as “I” for the defense‐inducer and “S” for the defense‐suppressor, and “U” for uninfested plants, and the outbred population is denoted as “O”.

The fecundity of the field and outbred populations of *T. urticae* were not significantly different in plants either with or without JA defenses (WT, *population*: $\chi_{3}^{2}$ = 0.910, *p* = 0.823, Figure 1A; *def‐1*, *population*: $\chi_{3}^{2}$ = 5.211, *p* = 0.157, Figure 1B), with all having a similar fecundity on WT and *def‐1* plants. Our benchmark controls followed a typical pattern of inducer and suppressor strains, respectively, with Santpoort‐2 having a lower fecundity on WT than Viçosa‐1, (*p* < 0.001), and both populations having similar fecundities on *def‐1* (*p* = 0.780).

### 3.2 Induction of Tomato Defense Genes by the Field‐Collected Spider Mites and Expression Levels of Their *Effector 84*

The normalized transcript levels of *effector 84* were different across populations (*population*: $\chi_{5}^{2}$: = 85.729, *p* < 0.001, Figure 1C). Post hoc comparisons revealed that these differences are due to high levels of expression of this gene in *T. evansi* Viçosa‐1, which are 50–150 times higher than for *T. urticae* . All *T. urticae* populations, including the defense‐inducer benchmark, had similar transcript levels of *effector 84*.

Four days post infestation (4 dpi), the normalized transcript levels of JA and SA marker genes were different among populations (*population*: *WIPI‐IIc*: $\chi_{6}^{2}$ = 49.805, *p* < 0.001; *WIPI‐IIf*: $\chi_{6}^{2}$ = 52.733, *p* < 0.001; *PR‐1a*: $\chi_{6}^{2}$ = 51.279, *p* < 0.001; Figure 1D–F). Transcript levels in leaflets infested with the defense‐suppressor benchmark were lower than the ones infested with the defense‐inducer benchmark. Transcript accumulation levels for *WIPI‐IIc* and *PR‐1a* in plants infested with the field‐collected or outbred populations were similar to the defense‐suppressor benchmark and some were even similar to uninfested plants (Outbred, ALP and MON for *WIPI‐IIc* and *PR‐1a*). The transcript levels of *WIPI‐IIf* in leaflets infested with MON were as high as for leaflets infested with the defense‐inducer benchmark.

### 3.3 Fecundity of the Biomarker Strain on Leaflets That Had Been Preinfested by Mites From Field‐Collected Populations

**FIGURE 2:**
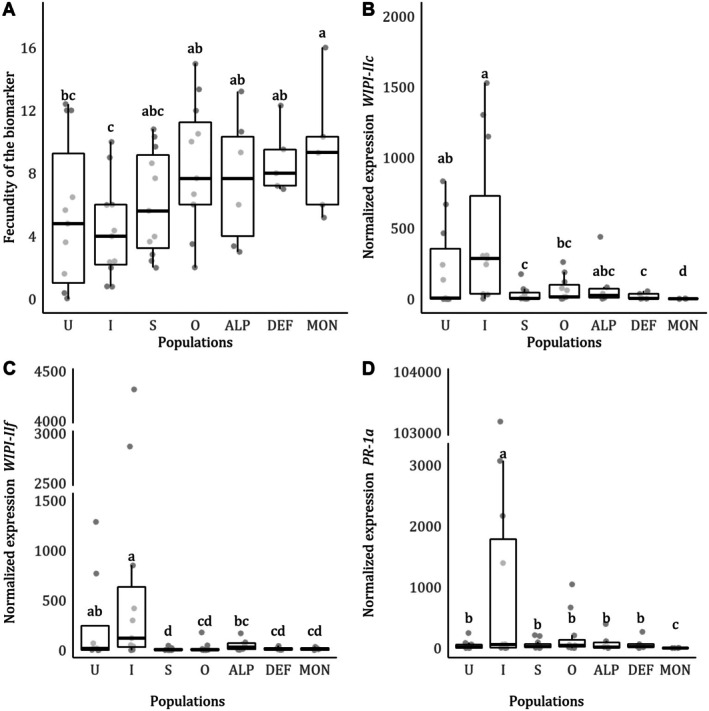
Effect of preinfestation on the biomarker. We measured (A) fecundity at 6 dpi and normalized gene expression of JA‐related genes (B) *WIPI‐IIc*, (C) *WIPI‐IIf*, and SA‐related defenses (D) *PR‐1a* at 6 dpi. Each circle represents individual data points (*n* = 5–11). Different lowercase letters denote statistical differences between populations according to multicomparison analysis performed using estimated marginal means. The benchmarks are denoted as “I” for the defense‐inducer and “S” for the defense‐suppressor, and “U” for uninfested plants, and the outbred population is denoted as “O.”

The population preinfesting the plant had a significant effect on the fecundity of the biomarker (*population*: $\chi_{6}^{2}$ = 23.843, *p* < 0.001; Figure 2A). Post hoc comparisons revealed that the biomarker had higher fecundity on plants preinfested with each of the four *T. urticae* populations tested (Outbred, ALP, DEF and MON; Figure 2A). However, there was no difference in fecundity on uninfested plants, or plants previously infested with the suppressor or the inducer benchmark.

After introducing three adult females of the biomarker strain, the transcript levels of all defense‐related genes changed (population*time point: WIPI‐IIc: $\chi_{6}^{2}$ = 35.944, *p* < 0.001; *WIPI‐IIf*: $\chi_{6}^{2}$ = 49.365, *p* < 0.001; *PR‐1a*: $\chi_{6}^{2}$ = 13.929, *p* = 0.030; Figure 2B–E). Post hoc comparisons revealed that for all genes, except for *WIPI‐IIc*, transcript levels of uninfested plants were higher at 6 dpi compared to 4 dpi. However, for plants preinfested with the field‐collected, outbred and benchmark populations, transcript levels mostly remained similar to, or were lower than, 4 dpi (lower at 6 dpi for (i) defense‐inducer benchmark, DEF and MON for *WIPI‐IIc*; (ii) defense‐suppressor, Outbred and MON for *WIPI‐IIf*; (iii) Outbred for *PR‐1a*).

### 3.4 Phylogenetic Analysis of *COI* and *Effector 84*

Twenty mitochondrial haplotypes (M1–M20) were found based on the *COI* sequences. All the populations sequenced, except Santpoort‐1, belong to the same lineage of *T. urticae* (Figure S3).

**FIGURE 3:**
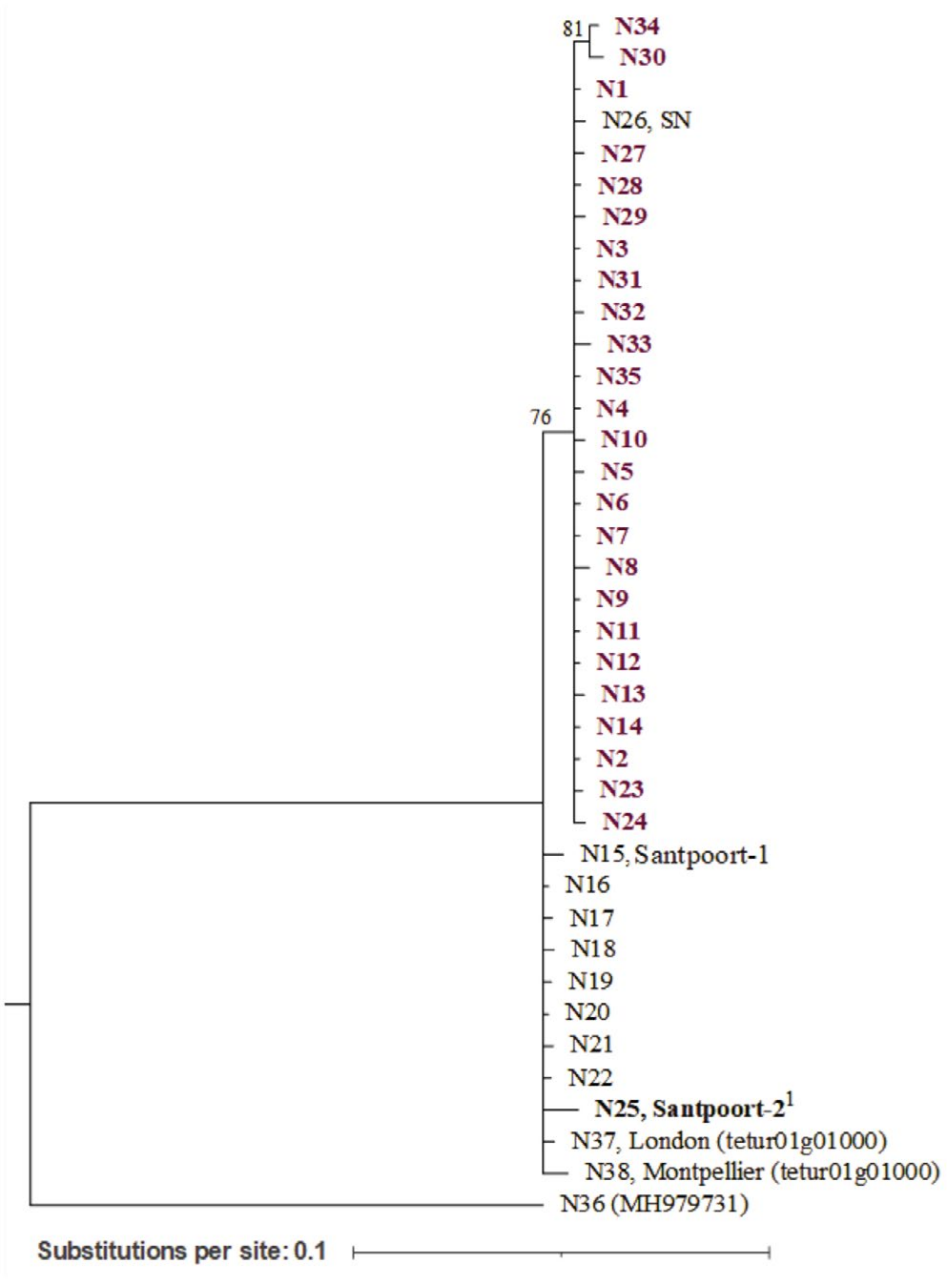
Phylogenetic relationships of *T. urticae* *effector 84* alleles (partial ORF sequence, 655 bp; 658 bp for *T. evansi* outgroup, N36). Alleles belonging to the populations phenotypically characterized in this study are highlighted in bold. Alleles from defense suppressor populations are highlighted in purple. Populations for which only one allele was found are referred to in front of the respective allele. Reference sequences have their accession numbers in brackets. For allelic codes see Table 1 (or Table S2 for more detail) and for SNPs see Figure S4. ^1^Defense‐inducer benchmark and biomarker strain.

For the *effector 84* (Figure 3), 38 alleles (N1–N38) were identified. The haplotype diversity (*H*_d_) and nucleotide diversity (*π*) of all sequences were 0.910 and 0.017, respectively (Table 2). From the populations harboring different alleles, DEF and the outbred populations were the ones with higher *H*_d_ and *π* (Table 2).

**TABLE 2:** *COI*‐haplotype, *effector 84* alleles and protein sequences codes, and measures of DNA polymorphism within populations for *effector 84*.

Population	*COI*‐haplotype	*Effector 84* alleles	Protein	*S*	*Eta*	*h*	*H*_d_ (±SD)	*π %* (±SD)
All sequences	M1–M20	N1–N38	P1–P26	155	162	38	0.910 (±0.018)	0.017 (±0.004)
ALP	M6	N1–N6	P1–P4	4	4	6	0.718 (±0.128)	0.002 (±0.001)
DEF	M6	N2 N6–N14	P1 P4–P8	9	9	10	0.895 (±0.070)	0.006 (±0.001)
MON	M6	N2 N23 N24	P1 P16 P17	9	9	3	0.464 (±0.200)	0.005 (±0.002)
Outbred	M6	N1 N2 N23 N27–N35	P1 P16 P17–P23	9	9	12	0.890 (±0.073)	0.006 (±0.001)
Santpoort‐1	M11	N15	P9	0	0	0	0	0
Santpoort‐2^a^	M13	N25	P18	0	0	0	0	0
SN	M7	N26	P19	0	0	0	0	0
London_NL	M6	N16–N22	P10–P15	7	7	7	0.792 (±0.089)	0.003 (±0.001)

*Note:* The number of polymorphic/segregating sites (*S*), the total number of mutations (*Eta*), the number of haplotypes (*h*), the haplotype diversity (Hd, Nei 1987), and the nucleotide diversity (*π*, Tajima 1983).

Abbreviation: NA, nonapplicable.

^a^ Defense‐inducer benchmark and biomarker strain.

All the individuals sequenced from the defense‐inducer population (Santpoort‐2) had one allele (N25), sharing the same phylogenetic clade with Santpoort‐1 (also with one allele, N15) and London_NL (with seven alleles). These populations shared the same clade with the reference alleles (N37 and N38). The population collected in Spain, SN (allele N26), and the Portuguese field and outbred populations were in a separate clade, supported by a bootstrap of 76%. The allele most shared between individuals in the field and, consequently in the outbred population, was N2 (represents 53% for ALP, 33% for DEF, 78% for MON, and 37% for Outbred of all allelic variations). We found multiple alleles within ALP, DEF and MON (5, 8, and 3 alleles, respectively) with some being singletons (i.e., alleles represented by a single sequence). Twelve alleles were found in the outbred population, of which 3 were also present in the field‐collected populations. The 38 alleles were found to encode 26 different proteins (P1–P26). The same arrangement of the phylogenetic tree for the nucleotide clades was observed for the protein sequences (Figure S3).

## 4 Discussion

In this study, we showed that spider mites collected from populations naturally occurring on tomato plants in the field can be classified as suppressors of tomato defenses. The field‐collected and outbred populations had similar fecundity on WT and *def‐1*, and they suppressed the induction of tomato defense genes by the defense‐inducer benchmark strain. Also, the field‐collected mites and the outbred population promoted the fecundity of the biomarker strain. Expression levels of *effector 84*, associated with defense suppression by mites, were similar across *T. urticae* lines that induce or suppress tomato defenses. We speculate that such allelic diversity may be subject to natural selection when these mites colonize a novel host plant and promote traits that counter plant resistance, such as defense suppression, thereby facilitating adaptation.

Our observation aligns well with predictions that follow previous research. Previous research showed that spider mites collected in the field from nonsolanaceous hosts, notably spindle tree and deadnettle, predominantly consist of individuals that are maladapted to tomato (as a novel host) (Kant et al. 2008; Alba et al. 2015). Most of these mites were found to strongly induce JA defenses in tomato plants and to be susceptible to these defenses (Kant et al. 2008; Alba et al. 2015). However, in these populations, individuals that can suppress or resist tomato defenses were occasionally found, albeit at low frequencies (Kant et al. 2008; Villacis‐Perez et al. 2022). This goes in line with what was previously shown (e.g., Magalhães et al. 2007), as it suggests that such natural spider mite populations may contain sufficient standing variation to adapt to a novel host like tomato. This was confirmed by Wybouw et al. (2015) where it was shown that spider mites from bean could adapt to tomato and that this coincided with the emergence of the ability to suppress tomato defenses. Together these observations suggest that spider mites living on a nontomato host (spindle tree, deadnettle, and castor oil) will have a lower fitness once on tomato but will adapt to it when natural selection favors the low‐frequency traits that allow these mites to resist or suppress tomato defenses.

### 4.1 Defense Suppression of Tomato Defenses Is Common in *T. urticae* Collected From Field‐Grown Tomatoes

The field populations showed similar oviposition on both hosts, suggesting that they can cope with induced JA defenses. As observed previously, defense‐inducer mites have lower oviposition on WT (Figure 1A) than suppressor mites (Kant et al. 2008; Alba et al. 2015), while in *def‐1* (Figure 1B) oviposition is similar for both populations (Li et al. 2002; Kant et al. 2008; Alba et al. 2015). However, similar levels of oviposition on both WT and *def‐1* can be a sign of either metabolic resistance against plant defenses induced by the spider mites or defense suppression (Kant et al. 2008).

We observed that all mites collected from populations of field‐grown tomato plants tested could suppress tomato defenses to varying levels and that this trait was maintained in an outbred population. For all tomato defense genes measured, normalized transcript accumulation was higher in plants infested by the defense‐inducer benchmark and lower for the remaining populations (Figure 1D–F), sometimes to levels of uninfested plants (for *WIPI‐IIc* and *PR‐1a*). However, some variation in levels of defenses was observed, with some populations not differing from the defense‐inducer benchmark for some genes (i.e., DEF for *WIPI‐IIc* and *PR‐*1a, and MON for *WIPI‐IIf*). Indeed, plant marker gene variability can introduce genetic diversity within supposedly homogeneous populations, being crucial to assess the effect of induction and suppression in mite fecundity, though a biomarker strain. Despite this variation, the overall results indicated that the field populations upregulate defenses to much lower levels than the defense‐inducer benchmark; whereas, in turn, the inducer benchmark did not upregulate defenses anymore in plants preinfested with a field strain (Figure 2B–D). These data indicate that field‐collected mites can suppress inducible plant defenses.

### 4.2 Spider Mites Collected From Field‐Grown Tomatoes Promote Fecundity and Suppress Defense Genes Induced by the Biomarker Strain

Previous results on the effect of suppression on the fecundity of a biomarker strain suggested that defense suppression has a persistent effect after the removal of the inducer/suppressor mites (Kant et al. 2008; Alba et al. 2015; Godinho et al. 2016; de Oliveira et al. 2016; Schimmel et al. 2017, 2018). However, whether the lasting effect of suppression also includes sustained suppression of defense gene expression was not previously assessed (but see Teodoro‐Paulo et al. 2023). The results here suggest this to be the case. Similarly, it had been found previously that suppression of defense genes by the *T. urticae* sister species *T. evansi* is retained for at least 4 days (de Oliveira et al. 2019). It is possible that, because mite feeding and oviposition occur in the same location, a lasting effect of suppression by the adults provides their offspring with better food (Bruessow et al. 2010). As such, a longer‐lasting effect of defense suppression, especially for SA marker genes, could provide a beneficial environment for offspring development. Investigating this further will give insights into the mechanism (why it is lasting), function, and ecological costs (who benefits from it—offspring and/or competitors). This may help to formulate testable hypotheses regarding mite life history, community composition, and adaptation.

In line with the defense gene induction/suppression pattern, we found that preinfestation with mites collected from field‐grown tomatoes and the outbred population increased the fecundity of the biomarker strain (Figure 2A). Our results indicate that, although the magnitude of suppression was not uniform across the mite lines we tested, the effect on the fecundity of the biomarker was similar. In this experiment, the effect of the defense‐inducer and defense‐suppressor benchmarks on the biomarker strain were not significant, probably due to a large amount of variation in the uninfested control (Figure 2A). Previous studies observed clearer effects of the induction and suppression benchmarks on the biomarker strain (Kant et al. 2008; Alba et al. 2015; Godinho et al. 2016). However, other studies also failed to observe significant effects using this assay despite seeing the expected trends and this may be due to power (sample size) and timing (de Oliveira et al. 2016; Schimmel et al. 2017, 2018; Blaazer et al. 2018).

### 4.3 There Is Significant Genetic Variation in *Effector 84* Across *T. urticae* Populations

Despite the variation in tomato defense suppression among the field‐collected spider mites and outbred population, the expression levels of the salivary *effector 84* were similar to those of the defense‐inducer benchmark (Figure 1C). *effector 84* from both *T. urticae* and *T. evansi* downregulate defense gene expression in *N. benthamiana* upon transient overexpression (Villarroel et al. 2016) and it can suppress elicitor‐induced cell death in both *N. benthamiana* and tomato (Cui et al. 2022). Moreover, *effector 84* expression in *T. urticae* correlates negatively with the magnitude of induced defenses in tomato (Schimmel et al. 2017; Liu, Chafi, et al. 2020; Liu, Legarrea, et al. 2020). However, given our *effector 84* expression data, it is certain that this effector is not the only determinant of defense suppression by *T. urticae* . Probably, induction/suppression results from a complex interplay between mite effectors and elicitors (Cui et al. 2022; Villarroel et al. 2016; Iida et al. 2019; Jonckheere et al. 2016; Jonckheere et al. 2018), and sequence and expression variation therein. To this end, we explored effector sequence variation at the nucleotide and amino acid level and performed a phylogenetic analysis to search for relationships between sequence variation and phenotype.

Unlike İnak et al. (2019), which found two mitochondrial lineages of *T. urticae*, our analysis revealed three mitochondrial lineages (Figure S3). This might be explained by the inclusion of several *COI‐*haplotypes not considered in the previous phylogeny, such as the Portuguese and Dutch populations, and the use of different phylogenetic methodologies. We found that the three field‐collected populations, and hence the outbred population, are of the same *COI*‐haplotype as London_NL (haplotype M6, lineage I, Figure S3). These populations shared the same lineage (lineage I) as the Spanish population (SN, haplotype M7) and the defense‐inducer benchmark (Santpoort‐2, haplotype M13), although these two were more closely related. Santpoort‐1 belongs to a more distinct lineage (lineage II, haplotype M11, Figure S3). Although the field‐collected populations shared the same *COI*‐haplotype (at least in the fragment sequenced), they are from different origins and have different nuclear alleles (*effector 84*, discussed below), suggesting that these populations are genetically different.

Across all the mite lines we tested, we found 38 different alleles for *effector 84*, and these translated into 26 different proteins. We do not know if some of the amino acid variations we observed affect *effector 84* functionality or structure. The alpha‐fold structure prediction of *effector 84* has low confidence (see Uniprot (UniProt Consortium 2019): T1JPW1) hampering accurate assessment to estimate the effect of single amino acid changes on structure and function using bioinformatics alone, and thus functional studies as in Villarroel et al. (2016) or Cui et al. (2022) will be necessary to explore this further. Across the *T. urticae* lines and populations we sequenced, *effector 84* has a high haplotype diversity (0.910, Table 2) but a very low nucleotide diversity (0.017%, Table 2). Patterns similar to this global one were observed in several of the local populations (ALP, DEF, London). These patterns may be the result of the action of purifying selection and/or rapid population rebound following a drastic past demographic bottleneck (Grant and Bowen 1998). We identified 38 alleles across all the populations sequenced, with the field populations harboring 15 of these, and the outbred population 12 (of which, three were shared with the field populations). This suggests high genetic differentiation between the founders of the outbred population and the individuals analyzed here representing each of the three field populations. This high genetic differentiation by chance underscores the huge haplotype diversity in each of the three source populations. Nevertheless, the SNPs present in the most common allele, which is shared between field‐collected and outbred populations, N2 (which codes for protein P1), could explain defense suppression in these populations.

### 4.4 Inducer and Suppressor Mites Predominantly Cluster in Different *Effector 84* Clades

We observed that inducer and suppressor populations do have different alleles and proteins. However, these differences do not segregate completely into inducer and suppressor mites (Figure 3). Instead, the Spanish population, SN (allele N26, found in an alleged inducer population, Sato et al. 2014) clustered with the field and outbred populations collected in Portugal (alleles N1–14, N23–24, and N27–35). Also, *T. urticae* Santpoort‐1 (allele N15, characterized as a suppressor population in Kant et al. 2008) clustered with the defense‐inducer benchmark, Santpoort‐2, and with *T. urticae* London (allele from reference genome: N25 and alleles from London_NL: N16–22). This can be explained by various factors. First, the two clusters observed in the *effector 84* gene tree may represent different geographical origins of the populations, as those collected in Portugal (ALP, DEF, MON) and Spain (SN) clustered together. Second, this clustering could be related to host adaptation. The populations collected in Portugal and Spain were collected on *Solanum* hosts, while the others were collected from more distal hosts. It was previously observed that populations of *T. urticae* from bean can adapt via natural selection to tomato plants, increasing their metabolite detoxification potential and their ability to reduce the production of plant defense compounds (Wybouw et al. 2015). This suggests that defense suppression of tomato defenses may rapidly emerge as a result of the selection on tomato (Blaazer et al. 2018). Indeed, the field‐collected *T. urticae* populations all had been obtained from tomato plants (in the same geographic area) and were maintained in the laboratory on tomato ever since (Godinho et al. 2020). Also, the creation of the outbred population was performed entirely on tomato plants. Hence adaptation to tomato may have acted on (low frequency) traits that, coincidentally or not, allow for suppressing tomato defenses. While selection for direct resistance to tomato defenses may be characterized by rapid selection for detoxification genes (Dermauw et al. 2013), selection for suppression may depend on selection for potent effector genes. This could have implications for agricultural systems (especially in monoculture scenarios) where the ecological costs of suppression may be relatively low due to a lack of community diversity. Although R‐gene resistance breeding in plants (Keith and Mitchell‐Olds 2013) may provide a temporary solution, the mere existence of multiple effector alleles in all the populations and strains suggests that such plant resistance can be broken easily (Pilet‐Nayel et al. 2017). We advocate that searching for effector targets to design breeding schemes to remove them or to make them invulnerable to effector manipulation (Zaidi et al. 2018) could offer an alternative approach, potentially complementing sustainable crop protection measures.

### 4.5 To Resist or to Suppress

Our results do raise important questions regarding the outcome of adaptive processes of herbivores that have to cope with the novel defenses of novel hosts such as mites on tomato.

First, the question of what determines whether suppression or resistance is favored by natural selection. This will probably strongly depend on the standing variation in the founder population, which, in turn, is determined by selection (time), drift, and gene flow on the previous host. Subsequently, it will also be determined by the physiological costs, determined by the ease by which mites can degrade, modify, secrete, or sequester defensive metabolites will be different across different plant hosts (Yang et al. 2001), and the ecological costs of the novel host may be. Also, the efficiency of an herbivore's effectors on the novel host, determined by the absence/presence of suitable targets (susceptibility proteins) as well as of R‐genes, and the effector variants present in the population will determine the success of suppression as an adaptive strategy (Blaazer et al. 2018). Furthermore, also the presence of competitors will influence whether resistance may be favored over suppression because the latter is a common good (Kant et al. 2008; Sarmento, Lemos, Bleeker, et al. 2011; Alba et al. 2015) whereas the first is not. Finally, it will be determined by the ability of the local herbivore population to mitigate the ecological costs, as the sister species *T. evansi* actively excludes its competitor *T. urticae* from suppressed plant material via thick webbing production (Sarmento, Lemos, Dias, et al. 2011), suppression localization (Schimmel et al. 2017), and reproductive interference (Sato et al. 2014).

Second, there is the question of whether resistance and suppression are complementary traits that can coexist in populations adapted, or whether one will exclude the other, or whether one will follow the other. Also, this will probably be determined by the physiology and secondary chemistry of the novel host as well as gene flow between mite populations in the vicinity of different hosts. It was suggested that suppression of defenses via effectors may promote fitness less than direct resistance (i.e., may be less effective in counteracting plant defenses in absolute terms, yet may operate on a wider host range, Kant et al. 2015). In that view, suppression may be favored by selection first (Wybouw et al. 2015) and then later be replaced by resistance to mitigate the ecological costs of suppression (Blaazer et al. 2018). However, this is not in line with the observation that populations from all over the world of the tomato specialist *T. evansi* cope with tomato and other hosts (Paulo et al. 2018) via suppression (Knegt et al. 2020). Maybe this species lacks the necessary building blocks for evolving resistance, or its active exclusion of competitors from its feeding site (Sarmento, Lemos, Dias, et al. 2011; Schimmel et al. 2017; Sato et al. 2014) may buffer selection against suppression.

All these hypotheses need to be addressed in future research to gain a better understanding of how herbivore intraspecific variation, gene flow, host plant identity, and community composition determine the outcome of adaptive processes of herbivores on novel hosts, and the evolution of host races, for specialists and also for generalists living on a mosaic of plant environments.

## 5 Conclusions and Perspectives

Our results show that the ability to suppress tomato defenses in *T. urticae* is common among mites living on tomato in the field. We argue that this trait may emerge in populations that have dispersed from an unrelated host, given that the traits needed for suppressing tomato defenses are included in its standing variation as had been shown previously (Kant et al. 2008; Alba et al. 2015; Wybouw et al. 2015). The question remains: What determines direct resistance to tomato defenses to emerge and how does this interact with suppression? Rapid adaptation of mites to plant resistance can be of great concern and especially impact monocultures in agriculture. Since traits related to induction/suppression and resistance/susceptibility to a novel host may be retained (at low frequencies) populations adapted to nontomato hosts (Kant et al. 2008; Villacis‐Perez et al. 2022), it would now be informative to transfer our tomato lines to unrelated hosts to see whether a similar adaptive response can be observed under these new conditions. We feel that studies regarding effector genetic and functional variation may reveal some of the mechanisms that drive plant–herbivore coevolution.

## Author Contributions

**Jéssica Teodoro‐Paulo:** conceptualization (lead), data curation (lead), formal analysis (equal), funding acquisition (equal), investigation (lead), methodology (lead), visualization (lead), writing – original draft (lead), writing – review and editing (equal). **Carlos Fernandes:** data curation (equal), formal analysis (equal), methodology (equal), software (equal), supervision (equal), validation (equal), writing – review and editing (equal). **Lin Dong:** investigation (supporting), methodology (supporting). **Sara Magalhães:** conceptualization (supporting), funding acquisition (equal), resources (equal), writing – review and editing (supporting). **Alison B. Duncan:** conceptualization (equal), data curation (equal), formal analysis (supporting), supervision (equal), validation (equal), writing – review and editing (equal). **Juan M. Alba:** conceptualization (equal), data curation (equal), formal analysis (equal), methodology (equal), supervision (equal), validation (equal), visualization (equal), writing – review and editing (equal). **Merijn R. Kant:** conceptualization (equal), data curation (equal), funding acquisition (lead), methodology (equal), resources (lead), software (lead), supervision (lead), validation (equal), visualization (equal), writing – original draft (equal), writing – review and editing (equal).

## Disclosure

Benefit‐Sharing Statement: Benefits from this research accrue from the sharing of our data and results on public databases as described above.

## Ethics Statement

The authors have nothing to report.

## Conflicts of Interest

The authors declare no conflicts of interest.

## Supporting information


Appendix S1

## Data Availability

Data are available in an open‐access data repository: https://doi.org/10.5061/dryad.j6q573nq9. Unique haplotype data are deposited to NCBI Nucleotide Database (OQ472023–OQ472057, OQ510005–OQ510008).
