# Supplementary material for: Suppression of Plant Resistance May Be a Common Trait Among Adapted Herbivores
Source: Ecol Evol. 2025 Jul 14;15(7):e71706. doi: 10.1002/ece3.71706 (PMC12259305; doi:10.1002/ece3.71706)
Supplement: Supplementary file 1 — Appendix S1 [file ECE3-15-e71706-s001.docx]

## Supplementary materials

Table S1. **Nucleotide sequences of primers used for qRT-PCR analysis and DNA amplification (adapted from Alba et al., 2015 and Villarroel et al., 2016).**

| Analysis | Target organism | Target gene | Name | Gene Identifier | Forward Primer  5’ 🡪 3’ | Reverse Primer  5’🡨3’ | References |
| --- | --- | --- | --- | --- | --- | --- | --- |
| qRT-PCR | *Solanum lycopersicum* | *WIPI-IIc* | *Wound-Induced Proteinase Inhibitor IIc* | Solyc03g020050.2.1 | CAGGATGTACGACGTGTTGC | GAGTTTGCAACCCTCTCCTG | Alba *et al.* 2015 |
|  |  | *WIPI-IIf* | *Wound-Induced Proteinase Inhibitor IIf* | Solyc03g020080.2.1 | GAGTGATGAACCCAAGGAGA | GTACAACACATATTATTAATGCATCA | Alba *et al.* 2015 |
|  |  | *PR-1a* | *Pathogenesis-related protein 1a* | Solyc09g007010.1.1 | TGGTGGTTCATTTCTTGCAACTAC | ATCAATCCGATCCACTTATCATTTTA | Alba *et al.* 2015 |
|  |  | *Actin* | *Actin* | Solyc03g078400.2.1 | TTAGCACCTTCCAGCAGATGT | AACAGACAGGACACTCGCACT | Alba *et al.* 2015 |
|  | *Tetranychus evansi* | *Te84* | *Tetranychus evansi secreted protein 84* | KT182961 | AACAAATGATTGGTGGCCTTG | TTCGAACAATTTACCGGATGC | Villarroel *et al.* 2016 |
|  | *Tetranychus urticae* | *Tu84* | *Tetranychus urticae secreted protein 84* | tetur01g01000 | TCTCAGTTGGTGGTGCTTTC | CGTTCATGGCATTGTCAAGG | Villarroel *et al.* 2016 |
|  | *Tetranychus evansi and Tetranychus urticae* | *RP49* | *Ribosomal protein 49* | tetur18g03590  Tevan_rep_c13981 | CTTCAAGCGGCATCAGAGC | CGCATCTGACCCTTGAACTTC | Villarroel *et al.* 2016 |
| DNA amplification | *Tetranychus urticae* | *COI* | *Cytochrome oxidase 1* | EU345430.1 | GGTCAACAAATCATAAAGATATTGG | TAAACTTCAGGGTGACCAAAAAATCA | Folmer *et al.* 1994 |
|  | *Tetranychus urticae* | *Tu84* | *Tetranychus urticae secreted protein 84* | tetur01g01000 | ATGAAGTCATTTTTAATTGTGATAGC | TTAGGCTGATTTTTCAACATCT | Villarroel *et al.* 2016 |

Table S2. **Nucleotide and amino acid sequences for the alleles of the nuclear gene *effector 84*.**

| **Nucleotide code** | **Amino acid**  **code** | **Number**  **of sequences** | **Population** | **ID** | **Reference**  **(GenBank accession numbers)** |
| --- | --- | --- | --- | --- | --- |
| N1 | P1 | 3 | ALP | 1 ,8 | OQ472023 |
|  |  |  | Outbred | 8 |  |
| N2 | P1 | 25 | ALP | 1(b), 2, 7, 9, 10, 12, 17 | OQ472024 |
|  |  |  | DEF | 10, 15, 18, 24, 28 |  |
|  |  |  | MON | 3, 4, 6, 7, 8(b), 9, 10 |  |
|  |  |  | Outbred | 4, 6, 9, 14, 15, 16 |  |
| N3 | P2 | 1 | ALP | 8(b) | OQ472025 |
| N4 | P2 | 1 | ALP | 9(b) | OQ472026 |
| N5 | P3 | 1 | ALP | 19 | OQ472027 |
| N6 | P4 | 3 | ALP | 19(b) | OQ472028 |
|  |  |  | DEF | 12 |  |
|  |  |  | DEF | 21(b) |  |
| N7 | P5 | 1 | DEF | 12(b) | OQ472029 |
| N8 | P6 | 1 | DEF | 17 | OQ472030 |
| N9 | P3 | 1 | DEF | 19 | OQ472031 |
| N10 | P7 | 1 | DEF | 19(b) | OQ472032 |
| N11 | P3 | 1 | DEF | 21 | OQ472033 |
| N12 | P7 | 1 | DEF | 22 | OQ472034 |
| N13 | P3 | 1 | DEF | 22(b) | OQ472035 |
| N14 | P8 | 1 | DEF | 25 | OQ472036 |
| N15 | P9 | 1 | Santpoort-1 | 2, 3, 6, 7, 9, 10, 25, 29 | OQ472037 |
| N16 | P10 | 8 | London_NL | 2, 6, 7, 18, 20, 21, 23(b), 27 | OQ472038 |
| N17 | P11 | 3 | London_NL | 18(b), 21(b), 23 | OQ472039 |
| N18 | P12 | 1 | London_NL | 20(b) | OQ472040 |
| N19 | P13 | 1 | London_NL | 22 | OQ472041 |
| N20 | P14 | 1 | London_NL | 22(b) | OQ472042 |
| N21 | P15 | 1 | London_NL | 24 | OQ472043 |
| N22 | P10 | 2 | London_NL | 24(b), 25 | OQ472044 |
| N23 | P16 | 2 | MON | 5 | OQ472045 |
|  |  |  | Outbred | 19 |  |
| N24 | P17 | 1 | MON | 8 | OQ472046 |
| N25 | P18 | 7 | Santpoort-2^1^ | 4, 6, 7, 15, 23, 24, 27, 28 | OQ472047 |
| N26 | P19 | 13 | SN | 1, 6, 7, 9, 11, 14, 16, 20, 23, 24, 27, 28, 30 | OQ472048 |
| N27 | P17 | 1 | Outbred | 8(b) | OQ472049 |
| N28 | P1 | 1 | Outbred | 9(b) | OQ472050 |
| N29 | P20 | 1 | Outbred | 13 | OQ472051 |
| N30 | P21 | 1 | Outbred | 13(b) | OQ472052 |
| N31 | P16 | 1 | Outbred | 17 | OQ472053 |
| N32 | P22 | 1 | Outbred | 17(b) | OQ472054 |
| N33 | P2 | 1 | Outbred | 18 | OQ472055 |
| N34 | P23 | 1 | Outbred | 18(b) | OQ472056 |
| N35 | P22 | 1 | Outbred | 19(b) | OQ472057 |
| N37 | P25 | 1 | London |  | Grbić *et al.* 2011  (OrcAE: tetur01g01000) |
| N38 | P26 | 1 | Montpellier |  | Grbić *et al.* 2011  (OrcAE: tetur01g01000) |
| N36 | P24 | 1 | *T. evansi* |  | Huang *et al.* 2019  (MH979731) |

^1^Defence-inducer benchmark and biomarker strain

Table S3. **Haplotype codes for the mitochondrial gene** ***COI*.** NA – non-applicable; NI – no information available.

| **Haplotype code** | **Number of sequences** | **Morph** | **Population** | **ID** | **Location** | **Field Host plant** | **GenBank accession numbers** | **Reference** |
| --- | --- | --- | --- | --- | --- | --- | --- | --- |
| M1 | 4 | red | TuGR |  | Egion,  Greece | Citrus limon | AJ316597 | Navajas *et al.* 2003 |
|  |  | red | Strain 3 |  | Antalya,  Turkey | Capsicum sp. | MK508714 | İnak *et al.* 2019 |
|  |  | red | Strain 4 |  | Antalya,  Turkey | Eggplant | MK508715 | İnak *et al.* 2019 |
|  |  | red | Strain 10 |  | Antalya,  Turkey | Solanum melongena | MK508721 | İnak *et al.* 2019 |
| M2 | 1 | green | TuS |  | Valencia,  Spain | Citrus aurantium | AJ316599 | Navajas *et al.* 2003 |
| M3 | 1 | green | TuET |  | Cairo,  Egypt | Convolvulus arvensis | AJ316605 | Navajas *et al.* 2003 |
| M4 | 1 | red | TuTN |  | Sousse,  Tunisia | Malva sp. | AJ316606 | Navajas *et al.* 2003 |
| M5 | 1 | green | TuI |  | Palermo,  Italy | Citrus limon | AJ316598 | Navajas *et al.* 2003 |
| M6 | 50 | **green** | **ALP** | **1, 2, 7, 8, 9, 10, 12, 17, 19, 20** | **Alpiarça, Portugal** | **Solanum lycopersicum** | **OQ510005** | **This study** |
|  |  | **green** | **DEF** | **10, 12, 15, 17, 18, 19, 21, 22, 24, 25, 28** | **Lisboa, Portugal** | **Solanum lycopersicum** | **OQ510005** | **This study** |
|  |  | **green** | **MON** | **1, 3, 6, 7, 8, 9, 10** | **Montemor-o-Novo, Portugal** | **Solanum lycopersicum** | **OQ510005** | **This study** |
|  |  | **green** | **Outbred** | **4, 6, 9, 13, 14, 15, 16, 18, 19** | **NA** | **NA** | **OQ510005** | **This study** |
|  |  | **green** | **London_NL** | **2, 7, 18, 20, 21, 22, 23, 24, 27** | **Ontario, Canada^1^** | **NI** | **OQ510005** | **This study** |
|  |  | green | T. urticae green |  | Quanzhou,  Japan | Citrullus  lanatus | KJ729022 | Chen *et al.* 2014 |
|  |  | green | Strain 7 |  | Antalya,  Turkey | Phaseolus vulgaris | MK508718 | İnak *et al.* 2019 |
|  |  | green | GSS |  | Germany | NI | MK508722 | İnak *et al.* 2019 |
| M7 | 17 | green | LS_VL |  | Ghent,  Belgium | Rosa sp. | EU345430 | Van Leeuwen *et al.* 2008 |
|  |  | green | BR_VL |  | selected from BR_VL | NA | EU556754 | Van Leeuwen *et al.* 2008 |
|  |  | green | Algarrobo-1 |  | Andalucía, Spain | Solanum nigrum | KF887952 | Alba *et al.* 2015 |
|  |  | **green** | **SN** | **3, 6, 7, 8, 9, 10, 14, 16, 20, 23, 24, 27, 28, 30** | **Málaga, S**  **pain** | **Solanum nigrum** | **OQ510006** | **This study** |
| M8 | 1 | green | 12_1 |  | Perpignan,  France | Datura stramonium | HM565893 | Mendonça *et al.* 2011 |
| M9 | 1 | green | 116_2 |  | La Gaude-Bord du Var,  France | Urtica  dioica | HM565896 | Mendonça *et al.* 2011 |
| M10 | 4 | green | 120_1 |  | Nice,  France | Phaseolus vulgaris | HM565897 | Mendonça *et al.* 2011 |
|  |  | green | 43_1 |  | S. Andreu de Llavaneres,  Spain | Solanum nigrum | HM565901 | Mendonça *et al.* 2011 |
|  |  | green | 97_1 |  | Santiago del Teide,  Canary Island | Musa acuminata | HM565902 | Mendonça *et al.* 2011 |
|  |  | green | 175_1 |  | Recife,  Brazil | Carica  papaya | HM565908 | Mendonça *et al.* 2011 |
| M11 | 11 | green | 41_2 |  | Vilademuls,  Spain | Solanum lycopersicum | HM565898 | Mendonça *et al.* 2011 |
|  |  | red | Houten-1 |  | Santpoort,  Netherlands | Euonymus europea | KF447572 | Alba *et al.* 2015 |
|  |  | **red** | **Santpoort-1** | **2, 3, 6, 9, 10, 24, 25, 28, 29** | **Santpoort,**  **Netherlands** | **Euonymus europea** | **OQ510007** | **This study** |
| M12 | 1 | green | 13_1 |  | Ieraptera,  Greece | Solanum melongena | HM565907 | Mendonça *et al.* 2011 |
| M13 | 11 | green | Santpoort-2 |  | Santpoort, Netherlands | Euonymus europea | KF447571 | Alba *et al.* 2015 |
|  |  | **green** | **Santpoort-2^2^** | **1, 3, 4, 6, 7, 15, 23, 25, 27, 28** | **Santpoort, Netherlands^3^** | **Euonymus europea** | **OQ510008** | **This study** |
| M14 | 1 | green | DeLier-1 |  | Santpoort, Netherlands | Euonymus europea | KF447574 | Alba *et al.* 2015 |
| M15 | 1 | red | T. urticae red |  | Kunming,  Japan | Solanum lycopersicum | KJ729023 | Chen *et al.* 2014 |
| M16 | 1 | green | SS |  | NI | NI | MF152824 | Sharma *et al.* 2019 |
| M17 | 1 | green | RR |  | NI | NI | MF152825 | Sharma *et al.* 2019 |
| M18 | 1 | red | Strain 1 |  | Mersin,  Turkey | Solanum melongena | MK508712 | İnak *et al.* 2019 |
| M19 | 5 | red | Strain 2 |  | Mersin,  Turkey | Cucurbita pepo | MK508713 | İnak *et al.* 2019 |
|  |  | red | Strain 5 |  | Antalya,  Turkey | Solanum melongena | MK508716 | İnak *et al.* 2019 |
|  |  | red | Strain 6 |  | Antalya,  Turkey | Solanum melongena | MK508717 | İnak *et al.* 2019 |
|  |  | red | Strain 8 |  | Antalya,  Turkey | Phaseolus vulgaris | MK508719 | İnak *et al.* 2019 |
|  |  | red | Strain 9 |  | Antalya,  Turkey | Cucurbita pepo | MK508720 | İnak *et al.* 2019 |
| M20 | 1 |  | T. evansi Viçosa-1 | Outgroup | Brazil | Solanum lycopersicum | KF447575 | Alba *et al.* 2015 |

^1^maintained in the lab in Amsterdam since 2013; ^2^ Defence-inducer benchmark and biomarker strain; ^3^ maintained in the lab in Portugal since 2018

| (A)  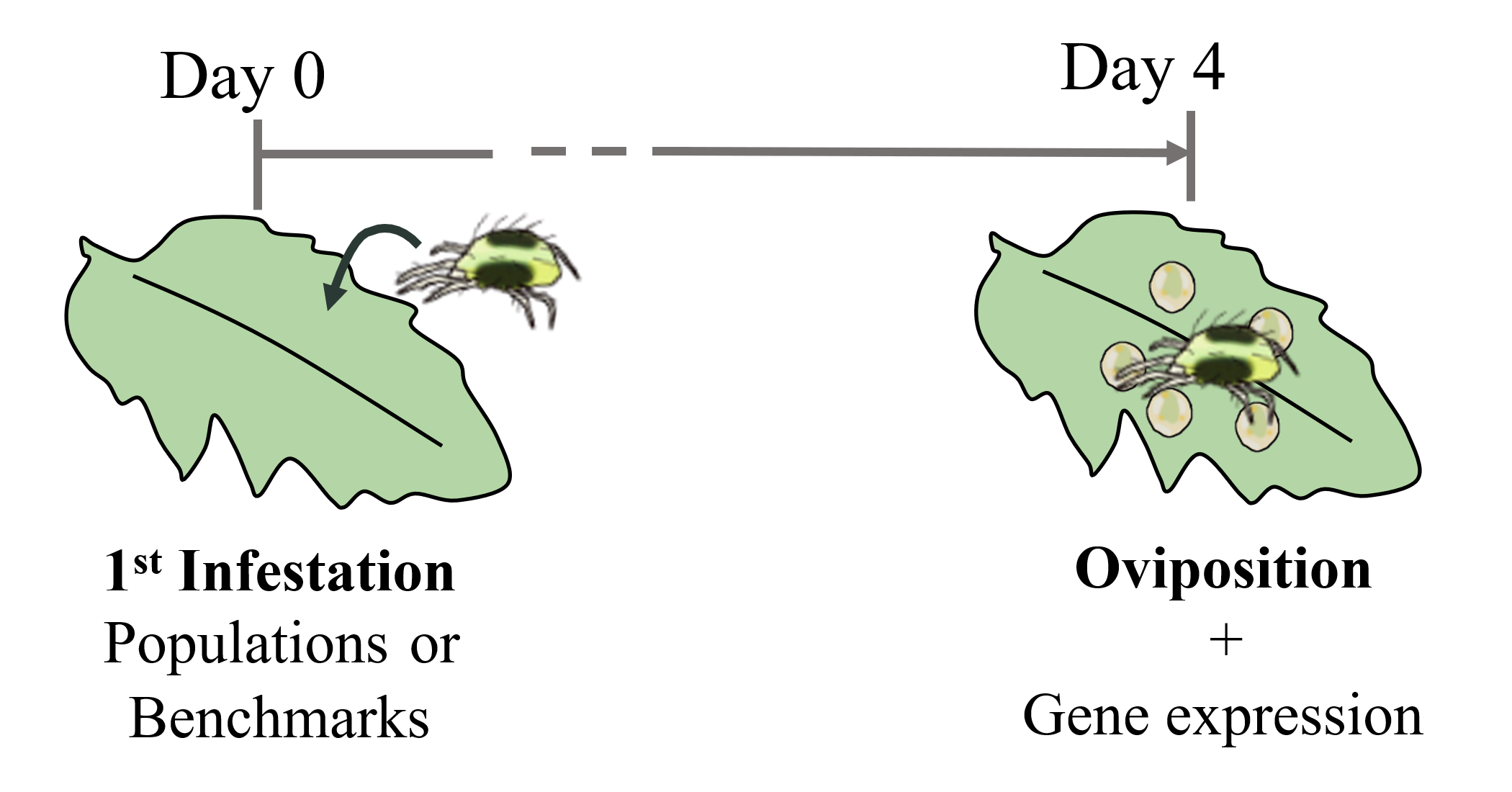 |
| --- |
| (B)  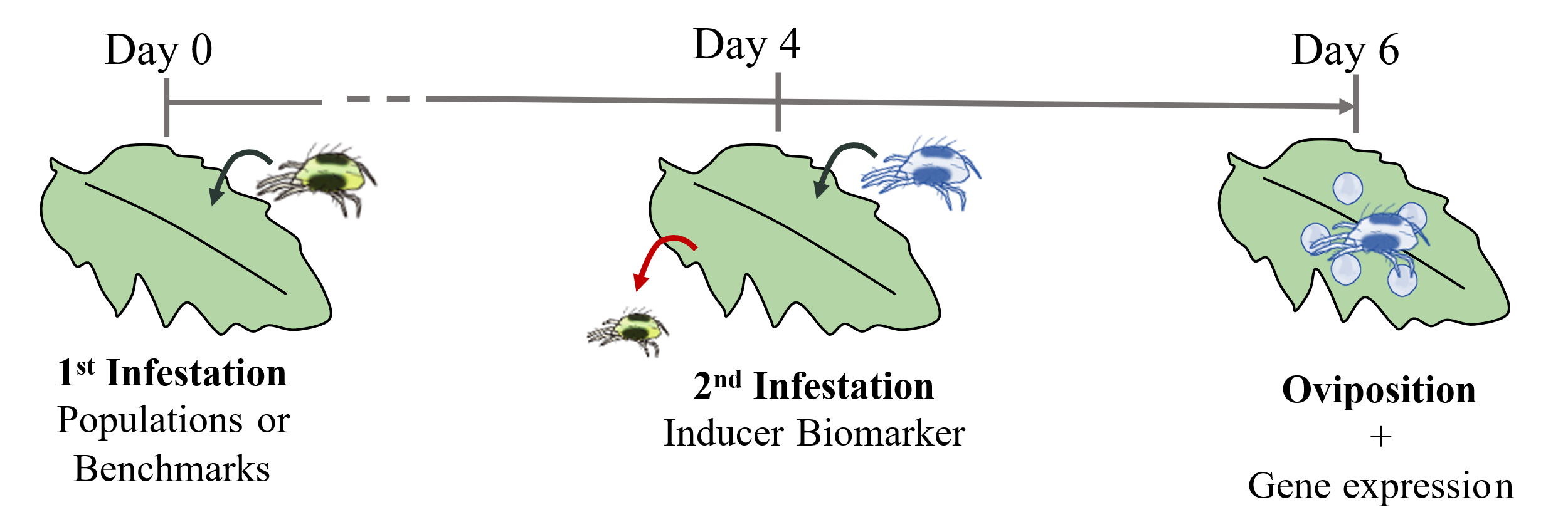 |

Figure S1 **Experimental setup.** (A) Fecundity of T. urticae from field-collected populations on WT and *def-1* (day 4), and (B) Fecundity of the biomarker strain on leaflets that had been pre-infested by mites from field-collected populations (day 6) and gene expression


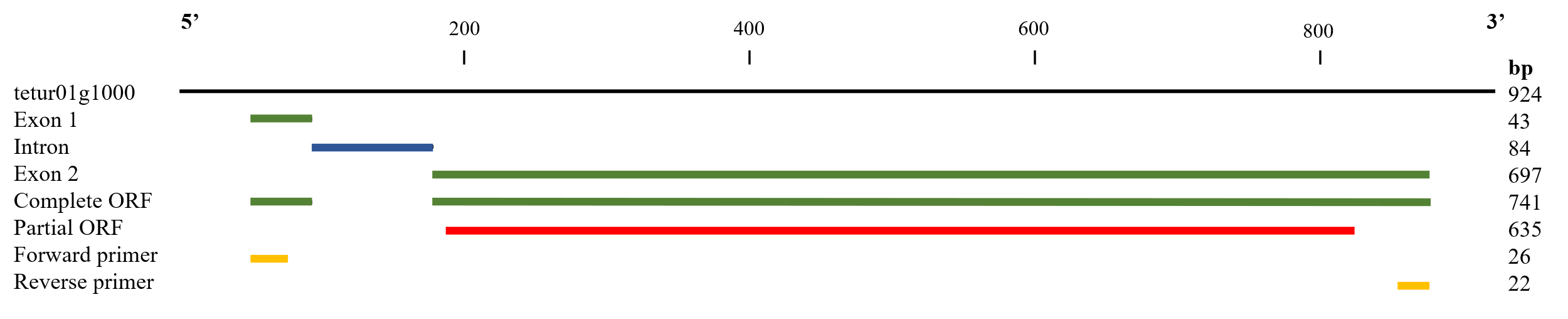


Figure S2 **Position of the partial ORF obtained in the gene model of *effector 84*.** tetur01g1000 in black represents the complete gene sequence. Both exons and the complete ORF are highlighted in green, and the primers used are represented in yellow. The intron is represented in blue. The partial ORF sequence (635 bp) used in this study is highlighted in red and aligns with the second exon.

Figure S3 **Phylogenetic relationship of *T. urticae* *COI*** (partial ORF sequence 385 bp). Haplotypes belonging to the populations sequenced in this study are highlighted in bold, with the name of the population referred to in front of the respective allele. ^1^Defence-inducer benchmark and biomarker strain. ^2^Defence-suppressor benchmark.

Figure S4 **Phylogenetic relationship of the partial amino acid sequence of effector 84 (221 amino acids; 222 for *T. evansi* outgroup).** Proteins belonging to the populations phenotypically characterized in this study are highlighted in bold. Proteins from suppressor populations are highlighted in purple. Populations for which only one protein variant was found are referred to in front of the respective protein code. Reference sequences have their gene accession numbers in brackets. For protein codes see Table 1 (or Table S2 for more detail). ^1^Defence-inducer benchmark and biomarker strain.
